# Functional cell permeable motifs within medically relevant proteins

**DOI:** 10.1016/j.jbiotec.2007.01.019

**Published:** 2007-05-01

**Authors:** Walter Low, Alison Mortlock, Liljana Petrovska, Tania Dottorini, Gordon Dougan, Andrea Crisanti

**Affiliations:** a Biological Sciences, Imperial College London, Imperial College Road, 5th floor SAF Building, London SW7 2AZ, UK; b Centre for Molecular, Microbiology and Infection, Imperial College London, London SW7 2AZ, UK; c Department of Experimental Medicine and Biochemical Science, University of Perugia, Perugia 06122, Italy; d The Wellcome Trust Sanger Institute, Wellcome Trust Genome Campus, Hinxton, Cambridge CB10 1SA, UK

**Keywords:** Toll-receptor 4 (TLR4), Toll-interleukin 1 receptor domain-containing adaptor protein (TIRAP), Protein transduction domains, TAT, Antennapedia homeodomain

## Abstract

Increasing experimental evidence indicates that short polybasic peptides are able to translocate across the membrane of living cells. However, these peptides, often derived from viruses and insects, may induce unspecific effects that could mask the action of their cargoes. Here, we show that a panel of lysine and/or arginine-rich peptides, derived from human proteins involved in cell signalling pathways leading to inflammation, possess the intrinsic ability to cross intact cellular membranes. These peptides are also capable of carrying a biologically active cargo. One of these peptides, encompassing the cell permeable sequence of the Toll-receptor 4 (TLR4) adaptor protein (TIRAP) and modified to carry a dominant-negative domain of the same TIRAP protein, selectively inhibited the production of pro-inflammatory cytokines upon LPS challenge, in in vitro, ex vivo and in vivo experiments. Docking studies indicated that this inhibition might be mediated by the disruption of the recruitment of downstream effector molecules. These results show for the first time the potential of using for therapy cell permeable peptides derived from human proteins involved in disease.

## 1 Introduction

The identification and characterisation of cell permeable peptides, or protein transduction domains (PTDs), has generated considerable interest, as they offer the opportunity to transport cargo polypeptides across cell membranes for a variety of applications. To date, PTDs have been used to deliver a number of molecules in vivo and in vitro, including enzymes, signalling molecules and apoptotic proteins, with the aim to dissect intracellular signalling pathways, regulate gene transcription, manipulate inflammatory responses and inhibit tumour progression (Lindsay, 2002; Christiaens et al., 2004; Wadia and Dowdy, 2005; Gondeau et al., 2005; Bernatchez et al., 2005; Toshchakov et al., 2005).

Among the inflammatory responses, those due to activation of Toll-like receptors (TLRs) have been intensively studied. A number of blocking peptides have been employed in vitro to delineate signal transduction pathways downstream of TLR4 and TLR2 (Horng et al., 2001; Toshchakov et al., 2005). Exposure of TLR4 to microbial components such as lipopolysaccharide (LPS) from Gram-negative bacteria triggers an intracellular signal cascade leading to the secretion of pro-inflammatory mediators followed by an immune response. TLRs share homologous cytoplasmic domains that are termed the Toll-Interleukin 1 receptor (TIR) homology domains. TIR domain-containing adaptors such as MyD88 (Medzhitov et al., 1998) and the TIR domain-containing adaptor protein (TIRAP) (Horng et al., 2001), also termed MyD88 adaptor-like (MAL) (Fitzgerald et al., 2001), couple TLR4 receptor activation to downstream signalling. These molecules are potential targets for therapeutic intervention and therefore it is anticipated that antagonists of TLR4 signal transduction might be able to modulate harmful pro-inflammatory responses such as those observed in septic shock or severe sepsis.

TIRAP was first identified by Horng et al. (2001) and its role in TLR4 signalling was investigated using a cell-permeable blocking peptide, encompassing a translocating sequence of the antennapedia homeodomain (Joliot et al., 1991; Derossi et al., 1994) covalently linked to a TIRAP dominant negative domain. This domain, termed the BB loop (Xu et al., 2000), is highly conserved among TIR domains. Horng et al. (2001) speculated that this peptide might act as a specific inhibitor of TIRAP by competing with TIRAP for interaction with TLR4. However, conflicting results obtained from studies carried out with macrophages from TIRAP knock-out mice (Yamamoto et al., 2002; Horng et al., 2002) have raised the possibility that inhibition with the TIRAP blocking-peptides may have been caused by the unspecific binding of the antennapedia transduction domain.

In addition to antennapedia, other functionally and structurally unrelated peptides, including TAT from human immunodeficiency virus (Green and Loewenstein, 1988; Frankel and Pabo, 1988) and VP22 from herpes simplex virus (Elliott and O’Hare, 1997), have been shown to cross intact cellular membranes. However, recent evidence suggests that, as in the case of antennapedia mentioned above, when used as carriers, these transducing peptides can cause unwanted effects that may either counteract the action of their cargoes or unspecifically enhance it (Fotin-Mleczek et al., 2005). Interestingly, in all cases, the ability to translocate maps to short amino acid sequences lacking in apparent sequence homology but characterised by a high content of basic amino acids such as arginine and lysine (Bogoyevitch et al., 2002; Vives, 2003). Substitution of arginine or lysine residues in TAT and antennapedia with alanines was shown to result in a marked decrease in peptide internalisation (Wender et al., 2000; Gratton et al., 2003).

In order to avoid the unspecific effects produced by ‘exogenous’ carriers such as Antenapedia, VP22 and TAT, we have investigated the cell-permeability properties of lysine and arginine-rich domains of human proteins which are known to be intimately involved in cell signalling pathways inherent to inflammation. A series of synthetic peptides was tested in vitro and shown to possess the intrinsic ability to move across cellular membranes. One of these peptides, derived from the TIRAP protein, was then fused to a cargo encompassing a dominant negative domain of the same protein. This fusion peptide, named TBX2, selectively delayed the kinetics of p38 activation following exposure to LPS in a fashion similar to that observed in cells from TIRAP knock-out mice. Furthermore, TBX2 selectively inhibited pro-inflammatory cytokine production upon LPS challenge in vivo. Docking analysis suggested that TBX2 may disrupt the heterotrimeric complex formation between TLR4, MyD88 and TIRAP by binding TLR4 at different non-overlapping sites. These results provide the rationale for the development of highly specific peptides for interfering with signal transduction and may pave the way to the development of novel tools for the treatment of human diseases such as sepsis syndrome and chronic inflammatory diseases.

## 2 Materials and methods

### 2.1 Database analysis

The EMBOSS (http://www.emboss.org) program fuzzpro was used to search SWISS-PROT for the following motifs [K/R]-X-[K/R]-[K/R]-[K/R], [K/R]-[K/R]-X-[K/R]-[K/R] and [K/R]-[K/R]-[K/R]-X-[K/R], where K is lysine, R is arginine and X is any amino acid. The resulting lists were parsed in Perl to produce a non-redundant list of entries.

### 2.2 Model building and computational methods

Initially, TBX2 secondary structure prediction was obtained using the PSIPRED v2.4 (Mcguffin et al., 2000) web-interfaced facilities [http://bioinf.cs.ucl.ac.uk/psipred]. The 3D model structure was constructed using the protein building module of Sybyl (version 7.0), and checked for atom and/or bond type correctness using the Sketch module of Sybyl. Hydrogen atoms were added using standard Sybyl geometries and minimized with Powell algorithm with a gradient of 1 kcal/(mol Å) and 1000 cycles to remove potential bad contacts. The peptide has been refined using RAPPER (Depristo et al., 2005). The final 3D structure present in the complex has been minimized using the Amber force field present in the compute module of Sybyl7.0.

### 2.3 Homology modelling

Sequence homologues were retrieved using PSI-BLAST (Altschul et al., 1997). Swiss-pdb (Guex and Peitsch, 1997) CPHModels2.0 server were used to generate 3D models of each protein. The models of the proteins have been obtained according to the PDB crystal structure coordinates of 1077 pdb (Tao et al., 2002). Evaluation of obtained structure values have been performed with the software WHAT IF http://biotech.embl-heidelberg.de:8400/) and RAMAPAGE (http://www-cryst.bioc.cam.ac.uk/rampage) and have been compared to the reference PDB. Hydrogen atoms were added to the models using standard Sybyl geometries, and minimized with the Powell algorithm with a gradient of 0.5 kcal/(mol Å) and 1000 cycles to remove potential bad contacts. Models have been analysed using the protein preparation tool in sybyl7.0.

### 2.4 Protein–protein, protein–peptide docking and HINT software

The models of complexes between TLR4 and the adaptor proteins, Tirap and Myd88, were constructed using the ClusPro Algorithm (Comeau et al., 2004). The HINT software (Kellogg et al., 1991) was used to predict protein–ligand, protein–protein and macrocycle–macrocycle associations by assessing hydrophobic, hydrogen bonds and polar interactions between the two interacting macromolecules. Protein–protein and protein–peptide complexes were minimized using the amber force field present is sybyl7.0.

### 2.5 Peptide synthesis

Peptides were produced by solid phase step-wise synthesis (SPSS) using the Fmoc N-terminal protection strategy. All peptides were made with C terminal carboxamide functionality. Chain assembly was performed on Applied Biosystems 433 or Pioneer automated synthesisers.

N terminal biotinylation was performed on the synthesiser through activation of the biotin carboxylic acid in the same way as for amino acids.

4-Chloro-7-nitrobenzofurazan (NBD) was attached to the peptide N terminal amino group using a manual method. This was achieved with high efficiency by incubating the N terminal deprotected peptide-resin with a five fold excess of NBD with two fold excess diisopropylethylamine (DIEA) in dimethylformaide (DMF) for 18 h with gentle mixing.

All peptide products were analysed by reverse phase HPLC or CZE as appropriate. Purification was performed by reverse phase HPLC. Confirmation of peptide molecular weight was performed by MALDI. All the peptides were solubilized in PBS.

Peptide sequences: **TAT**, GRKKRRQRRRPPQ; **TAT**^**Ala**^, GRKKAAQAAAPPQ; **IRAK1**, CLHRRAKRRPPMTQVYER; **TLR4**, GRHIFWRRLRKALLDGKSWNPE; **TIRAP**, GKMADWFRQTLLKKPKKRPNSPEST; **TIRAP**^**Ala**^, GKMADWFRQTLLAAPAAAPNSPEST; **BX2**, LQLRDATPGGAIVS; **TBX2**, GKMADWFRQTLLKKPKKRPNSPESTLQLRDATPGGAIVS; **Antp-TIRAP**, RQIKIWFQNRRMKWKKLQLRDAAPGGAIVS; **PU.l**, GSKKKIRLYQFLLDLLRSGDMKDS; **ICE**, QLLRKKRRIFIHSVGAGT.

### 2.6 Peptide internalisation assays

HeLa or RAW 264.7 cells were cultured at 37 °C, 10% CO_2_, in Dulbecco's modified Eagle's medium (DMEM) (Invitrogen), containing 10% foetal calf serum (FCS, Harlan), 100 U/ml penicillin and 100 μg/ml streptomycin. Quantification of internalisation of NBD-labelled peptides was carried out according to the procedure described by Drin et al. (2001). Briefly, cells were seeded in six-well plates (5 × 10^5^ cells/well) and treated with 0–160 μM of peptide for 2 h at 37 °C. Cells were washed twice in PBS, harvested using a solution of Versene (1:500) (Invitrogen) and re-suspended in 0.5 ml (final volume) of ice-cold PBS. Cells were analysed by flow cytometry using a Becton Dickinson FACS Scan instrument and the geometric mean of fluorescence intensity was considered to represent the amount of cell-associated peptide. Following flow cytometry, 5 μl of freshly prepared dithionite stock solution (1 M Na_2_S_2_O_4_ in 1 M Tris–HCl (pH 10)) was added to cells maintained at 4 °C for 5 min and analysed again with flow cytometry to measure residual fluorescence. Fluorescence from cells treated with dithionite only was subtracted from the fluorescence of cell-associated or internalised peptides to infer background values.

### 2.7 Immunoblot analysis

RAW 264.7 cells were treated with PBS or 160 μM TBX2 peptide for 2 h at 37 °C, 10% CO_2_, in DMEM, containing 10% foetal calf serum and antibiotics. Cells were stimulated with 10 ng/ml of LPS and samples were collected after 0, 10, 20 and 45 min. Cells were lysed in sample buffer and separated by 12% SDS-PAGE and transferred onto nitrocellulose membrane (Amersham). Membranes were blocked for 1 h in 5% dry milk in PBS and 0.1% Tween 20 at RT, and then probed with an anti-ACTIVE p38 (Promega) antibody for 1 h. After washing with PBS and 0.1% Tween 20, the primary antibody was detected using a goat anti-rabbit horseradish peroxidase conjugate (1:2000, Jackson Labs). To control that different samples were loaded in equal amounts, the membranes were also incubated with the mouse monoclonal anti-pan phospho-tyrosine antibody 4G10 (a gift from X. Montano) followed by a goat-anti mouse Horse Radish Peroxidase (HRPO) antibody (1:2000, Jackson Labs). Detection of HRPO antibodies was performed using an enhanced chemiluminescence detection system (ECL, Amersham).

### 2.8 Cytokine analysis

RAW 264.7 cells were treated with different concentrations of peptides for 2 h at 37 °C, 10% CO_2_, in DMEM, containing 10% foetal calf serum and antibiotics and then stimulated with 10 ng/ml LPS (*E. coli* 055:B5, Sigma), 25 μg/ml Poly I:C (InvivoGen) or 1 μg/ml R848 (InvivoGen). Supernatants were collected 22 h post agonist challenge and IL-6 levels quantified by ELISA, according to the manufacturer's instructions (R&D Systems). Primary human macrophages, isolated from a single donors buffy coats, were treated with different concentrations of the TBX2 or the BX2 peptides for 2 h at 37 °C, 10% CO_2_, in DMEM, containing 10% autologous serum and antibiotics and then stimulated with 1 ng/ml LPS for 18 h. The levels of TNF-α present in the supernatants were quantified by ELISA, according to the manufacturer's instructions (Pharmingen).

### 2.9 Animal experiments

Groups of five female mice (strain C3H/HeN) were injected intravenously (I.V.) with a single dose of TBX2 peptide (2, 4 or 6 mg per mouse) or PBS only. After 45 min, the mice were challenged with 10 μg of LPS injected i.v. 90 min later, the mice were anaesthetised and blood collected by cardiac puncture. The levels of TNF-α in the serum were quantified by ELISA according to the manufacturer's instructions (Pharmingen). All animal experiments were performed in compliance with institutionally approved protocols at Imperial College.

### 2.10 Cell viability

CellTiter-Glo Luminescent Assay (Promega) was utilised to determine viability of RAW 264.7 cells following treatment with peptides, according to the manufacturer's instructions.

### 2.11 Statistical analysis

Standard deviation (S.D.) and standard error of the mean (S.E.M.) were calculated using Microsoft Excel. Statistical analysis was carried out using Student's *t*-test.

## 3 Results

### 3.1 Peptides derived from human proteins and containing cationic motifs are capable of crossing the membrane of living cells

**Fig. 1:**
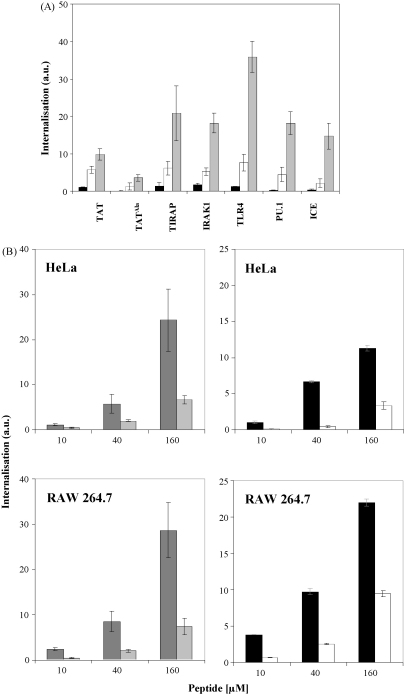
Internalisation of peptides. (A) Mean fluorescence values of NBD-treated HeLa cells measured after the addition of sodium dithionite quencher. Background values of cell treated with sodium dithionite only were subtracted. Cells (5 × l0^5^ cells ml^−1^) were incubated with 10 (black bar), 40 (grey bar) and 160 μM (white bar) of each of the NBD labelled peptides TAT (GRKKRRQRRRPPQ), TAT^Ala^ (GRKKAAQAAAPPQ), TIRAP (GKMADWFRQTLLKKPKKRPNSPEST), IRAKI (CLHRRAKRRPPMTQVYER), TLR4 (GRHIFWRRLRKALLDGKSWNPE), PU.1 (GSKKKIRLYQFLLDLLRSGDMKDS) and ICE (QLLRKKRRIFIHSVGAGT) for 2 h. The results are the average of three independent experiments. Error bars represent standard error of the mean (*n* = 3). (B) Structure to function analysis of the cell permeable motif found in TIRAP. Increasing concentrations of the NBD-labelled peptides TIRAP (dark grey bar), TIRAP^Ala^ (GKMADWFRQTLLAAPAAAPNSPEST) (light grey bars), TAT (black bars) and TAT ^Ala^ (white bars) were incubated with HeLa and RAW 264.7 cells. The amount of internalised peptide was determined by adding sodium dithionite quencher as described. The results are the average of three independent experiments. Error bars represent standard error of the mean (*n* = 3).

In addition to the well characterised polybasic PTDs (Frankel and Pabo, 1988; Green and Loewenstein, 1988; Joliot et al., 1991; Derossi et al., 1994; Elliott and O’Hare, 1997), a nuclear localisation signal (NLS) derived from SV40 and containing four lysines and one arginine (Peitz et al., 2002), and a peptide derived from the prion protein (PrP) containing the sequence KKRPK (Lundberg et al., 2002), were shown to be able to translocate across cell membranes. On the basis of these observations, we formulated the hypothesis that short stretches of cationic amino acids could confer cell permeable properties to certain human peptides. We therefore screened the human proteome databases for proteins containing motifs encompassing five charged residues of either arginine or lysine allowing for a single mismatch at positions 2, 3 or 4. A search in the Swiss-Prot protein database (http://us.expasy.org/sprot/) identified a large number of non-redundant, non-hypothetical human proteins known to function as kinases, transcription factors, proto-oncogenes, receptors and DNA repair enzymes. To test our hypothesis, focusing on cell signalling proteins linked to immune responses and inflammation, five peptides derived from proteins linked to pathological conditions and/or to cellular signalling pathways were synthesised and their ability to function as PTDs assessed. These included the human Toll-interleukin 1 receptor (TIR) domain-containing adapter protein (TIRAP) (Horng et al., 2001) (alternatively known as MyD88-adapter-like (Mal); Fitzgerald et al., 2001), the Interleukin-1 receptor-associated kinase 1 (IRAK1), the toll-like receptor 4 (TLR4), the transcription factor PU.1 (PU.1) and the caspase-1 inhibitor Iceberg (ICE). The peptides were designed to encompass the cationic motif flanked by endogenous sequences of up to 20 amino acids, and linked to a 7-nitrobenz-2-oxo-l,3-diazol-4-yl (NBD) at the amino terminal end (Drin et al., 2001). This fluorophore is irreversibly quenched by the addition of cell impermeable sodium dithionite, thus enabling discrimination of peptides merely bound to the cells from truly intracellular ones. Moreover, its use allows a direct measurement of the amount of internalised peptides in live cells, therefore avoiding artefacts due to fixation (Langeduk et al., 2004; Drin et al., 2003; Christiaens et al., 2004). Cultured epithelial HeLa cells were incubated with NBD-labelled peptides at three concentrations either in the presence or in the absence of sodium dithionite. Cultured cells were also treated with a TAT peptide and a mutated, TAT^Ala^ in which five arginine residues were substituted by alanines, to serve as positive and negative controls, respectively. Peptide internalisation was deduced by quantifying cell fluorescence by FACS analysis. All tested peptides gave fluorescence values similar or higher than TAT when added to HeLa cells in the presence of sodium dithionite, whereas very little fluorescence was observed with TAT^Ala^ at all concentrations tested. The fluorescent signal increased in a peptide concentration-dependent manner (Fig. 1A), in agreement with previous reports on TAT and Antp (Drin et al., 2003; Derossi et al., 1996; Oehlke et al., 1997; Hallbrink et al., 2001). The specificity of the uptake was investigated by incubating both murine macrophage-derived RAW 264.7 and HeLa cells either with the NBD-labelled TIRAP peptide, or with a mutated version in which the basic residues forming the cationic motif were replaced with alanines (TIRAP^Ala^) (Fig. 1B). Cells treated with NBD-TIRAP^Ala^ were substantially less fluorescent than those treated with the original TIRAP peptide, indicating that the disruption of the cationic motif abolished the ability of the peptide to cross the cell membrane.

### 3.2 The human-derived cell permeable peptides can carry a biologically active cargo

**Fig. 2:**
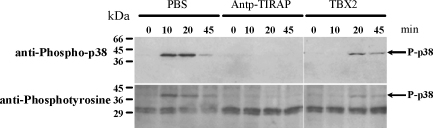
Kinetics of p38 phosphorylation in LPS treated RAW 264.7 macrophages upon addition of TBX2. Cells were pre-incubated with PBS, 40 μM of Antp-TIRAP (RQIKIWFQNRRMKWKKLQLRDAAPGGAIVS) or 160 μM of TBX2 (GKMADWFRQTLLKKPKKRPNSPESTLQLRDATPGGAIVS) for 2 h and then stimulated with 10 ng/ml LPS for the indicated periods of time. Cell lysates were separated on 12% SDS-polyacrylamide gel and blotted onto nitrocellulose membrane. Activation of p38 was detected using a specific anti-phospho-p38 antibody. For each experiment, the amount of cell lysate loaded was assessed using an anti-pan phospo-tyrosine antibody. Immunoblot analysis is representative of four independent experiments.

To assess the biochemical and biological activity of peptides containing putative PTDs in vitro and in vivo, we combined in a synthetic peptide the functional cell permeable sequence of TIRAP with a dominant negative domain from the same protein. This peptide, named TBX2, therefore encompassed the sequence of TIRAP shown to have cell permeable properties in HeLa and RAW 264.7 cells (Fig. 1B) (amino-acids 19–43) and a region of the BOX2 domain (Fitzgerald et al., 2001) (amino-acids 118–131), also known as the BB-loop (Xu et al., 2000). This region is highly conserved among TIR domains and forms the core of a dominant negative domain by competing the interaction of endogenous TIRAP with the cytoplasmic tail of Toll receptor 4 (TLR4) (Horng et al., 2001). To investigate whether the TIRAP dominant negative domain linked to the cell permeable sequence present in TBX2 was able to inhibit TLR4 activity, RAW 264.7 cells were treated with LPS and increasing concentrations of TBX2. The effect of TBX2 on the TIRAP-dependent activation of p38, a member of the mitogen activated protein kinase family, was assessed by immunoblot analysis using an antibody that selectively recognises the phosphorylated, active form of p38. As shown in Fig. 2, TBX2 caused a temporal shift in the appearance of the peak of phosphorylated p38 in RAW 264.7 cells upon LPS treatment. These results are in agreement with the observed kinetics of p38 phosphorylation in isolated peritoneal macrophages (Yamamoto et al., 2002) and bone marrow-derived macrophages (Horng et al., 2002) from TIRAP knock-out mice in response to LPS. Notably, the treatment with the Antp-TIRAP peptide, encompassing the PTD of Antp from *D. melanogaster* and the BB loop sequence derived from the mouse TIRAP gene (Horng et al., 2001), resulted instead in complete inhibition of p38 phosphorylation in response to LPS (Fig. 2). Previous reports had shown that the BB-loop, when fused to the PTD of Antennapedia, could inhibit the activation of MAP kinase JNK and NF-κB in RAW 264.7 cells upon LPS challenge (Horng et al., 2001). While TBX2 reproduced the p38 activation pattern observed in TIRAP knock-out mice, Antp-TIRAP did not.

### 3.3 Suppression of cytokine production following transduction of the TBX2 peptide in vitro, ex vivo and in vivo

**Fig. 3:**
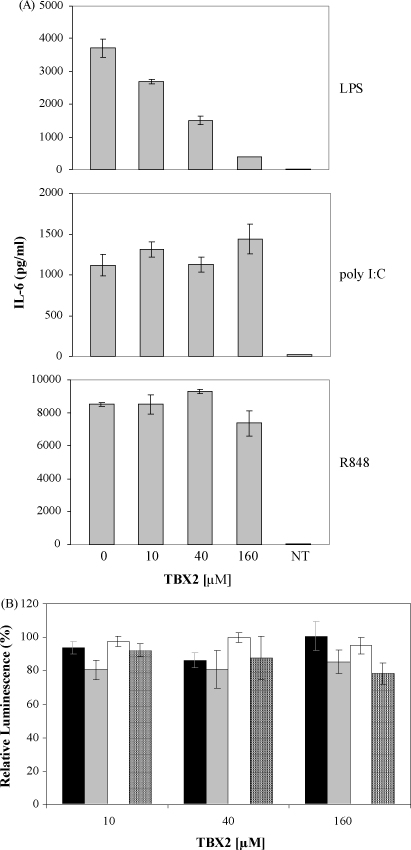
Activity of the TBX2 peptide. (A) TBX2 activity on IL-6 secretion of LPS stimulated RAW 264.7 cells. RAW cells were pre-incubated with increasing concentrations of TBX2 (GKMADWFRQTLLKKPKKRPNSPESTLQLRDATPGGAIVS) for 2 h and then challenged with 10 ng/ml LPS, or 25 μg/ml Poly I:C or 1 μg/ml R848. Supernatants were collected after 18 h and IL-6 levels quantified by ELISA. Results show one experiment carried out in triplicates representative of four independent experiments. Error bars represent standard error of the mean. NT: non-treated. (B) Cell viability assessed after incubation with TBX2. RAW 264.7 cells (l × 10^4^ cells ml^−1^) were distributed in 96-well plates and incubated for 2 (black bar), 4 (grey bar), 8 (white bars) and 18 h (grey dotted bars) with 10, 40 and 160 μM TBX2.

TIRAP-deficient mice show an impaired production of the inflammatory cytokine IL-6 in response to LPS (Yamamoto et al., 2002; Horng et al., 2002). Consistently, incubation of RAW 264.7 cells with TBX2 resulted in dose-dependent inhibition of IL-6 production in response to LPS (Fig. 3A).

To assess the specificity of TBX2-mediated inhibition on the TLR4-TIRAP signalling pathway, RAW 264.7 cells were treated with either Poly (I:C) (a ligand of TLR3) or the synthetic compound R-848 (a ligand of TLR7) (Fig. 3A). In either case, TBX2 did not inhibit IL-6 secretion. Furthermore, no significant reduction in cellular viability was observed following treatment with increasing concentrations of TBX2 over an incubation period of up to 18 h (Fig. 3B).

**Fig. 4:**
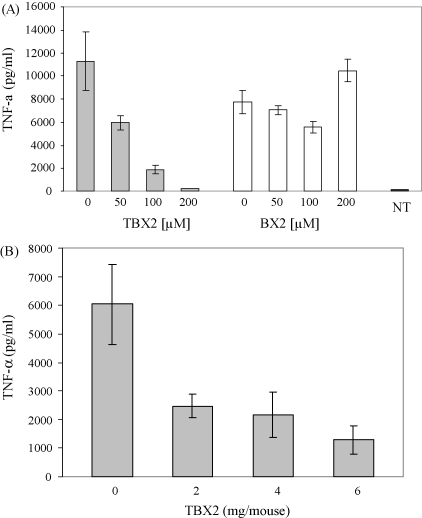
Activity of the TBX2 peptide ex vivo and in vivo. (A) TBX2 activity on TNF-α production of LPS stimulated primary human macrophages. Primary human macrophages (see Section 2) were pre- treated with increasing concentrations of either TBX2 (GKMADWFRQTLLKKPKKRPNSPESTLQLRDATPGGAIVS) (light grey bars) or BX2 (LQLRDATPGGAIVS) (white bars) for 2 h and then challenged with 1 ng/ml LPS for 20 h. TNF-α production of non-challenged and non-treated cells is shown (NT). TNF-α levels in the supernatants were quantified by ELISA. Results are the average of triplicates and representative of two independent experiments. Error bars represent standard error of the mean. (B) TBX2 activity on TNF-α response of LPS treated mice. Groups of five mice were injected intravenously (i.v.) with a single dose of TBX2 peptide (2, 4 or 6 mg per mouse) or PBS only. After 45 min, the mice were challenged with 10 μg of LPS injected i.v. 90 min later, the mice were anaesthetised and blood collected by cardiac puncture. TNF-α levels in the blood were determined by ELISA. Results are representative of two independent experiments. Levels of TNF-α measured on non-challenged and PBS-treated mice were subtracted from TNF-α values obtained from peptide treated mice. Error bars represents standard error of the mean *n* = 5. ^*^p < 0.05, ^**^p < 0.01.

We also assessed the ability of TBX2 to inhibit the production of TNF-α, a key mediator of chronic inflammatory diseases, in freshly isolated human macrophages. Our results indicated that at a 50 μM concentration of TBX2, TNF-α production from human macrophages was reduced by 50% in response to LPS (Fig. 4A). A 14 amino-acid peptide, BX2, encompassing only the TIRAP dominant negative sequence (Horng et al., 2001) and lacking the cell permeable domain, was used as control. As expected, primary human macrophages exposed to LPS produced similar levels of TNF-α either in the presence or absence of the BX2 peptide (Fig. 4A).

To determine whether TBX2 had the ability to suppress the expression of inflammatory cytokine genes in vivo, a single dose of the peptide was administered intravenously to mice 45 min prior to challenge with LPS. The levels of TNF-α, measured in ELISA on whole mice blood, were significantly reduced (over 60%) in mice treated with 2 mg of the TBX2 peptide when compared with control mice (Fig. 4B). A low level of IL-10 secretion was observed which did not differ significantly from animals treated with LPS only to animals treated with LPS and TBX2 (not shown).

### 3.4 TBX2 might interfer with the heterotrimeric complex formation between TLR4, MyD88 and TIRAP

**Fig. 5:**
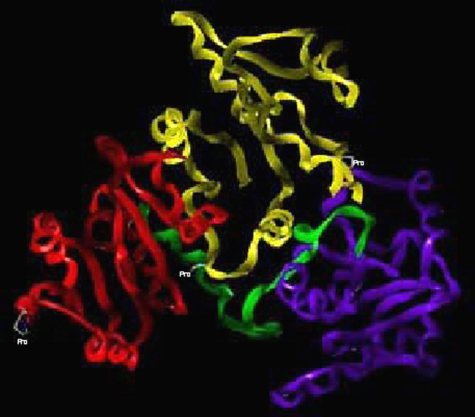
Molecular modelling and prediction of protein–protein interaction. TLR4 (yellow), TIRAP (purple), MyD88 (red) and TBX2 (green) are shown in ribbon representation. Conserved prolines of TLR4, MyD88 and TIRAP are shown in ball and stick. Non-overlapping binding sites for TIRAP and MyD88 on TLR4 and the hypothetical interactions between TBX2 and TLR4 are shown. The TBX2 associates via the BB loop with TLR4 on the TIRAP binding site and via its amino terminus (α helix) projected towards the region of TLR4 that is predicted to bind MyD88.

To obtain a better insight into the nature of TBX2 inhibition of p38 phosphorylation in response to LPS, we have modelled the TIR domains of TLR4, MyD88 and TIRAP proteins. The TIRAP and MyD88 adaptor proteins were docked onto the TLR4 using Cluspro (Comeau et al., 2004). Protein–protein complexes (TLR4-TIRAP and TLR4-MyD88) were superimposed to visualize the potential ternary complex associations. The models obtained were evaluated using the available biological information and post-processed using the HINT software (Kellogg et al., 1991). This software predicts protein–ligand, protein–protein and macrocycle–macrocycle associations by assessing hydrophobic, hydrogen bonds and polar interactions between the two interacting macromolecules. Our results suggest that TIRAP and MyD88 bind to TLR4 in two non-overlapping binding sites (Fig. 5). In this model, TIRAP is predicted to associate through its BB-loop to TLR4, whereas MyD88 is predicted to interact with the region enclosing the TLR4 BB-loop. The P714H mutation on TLR4 has been shown to disrupt MyD88/TLR4 complex formation (Poltorak et al., 1998) suggesting that this residue could be involved in the binding of Myd88. This is consistent with our model in which the BB-loop of TLR4 is directed towards MyD88. A corresponding proline residue is also found in the BB-loops of TIRAP and MyD88. Mutation of this proline (P125H) to histidine on the TIRAP protein was proposed (Horng et al., 2001) to disrupt TIRAP-TLR4 association. Accordingly, in our TLR4-TIRAP interaction model, mutation of this proline (P125H) to histidine could result in hydrophobic clashes. In addition, Dunne et al. (2003) showed that the MyD88 P200 mutation has no effect on TLR4-MyD88 association. Again, this is consistent with our model in which the BB-loop of MyD88 is projected on the opposite side of TLR4.

We then considered this ternary complex model as a basis to determine whether TBX2 could occupy crucial sites involved in protein–protein interaction with TLR4. Thus, we performed peptide–protein docking (TBX2-TLR4) analysis using TBX2 as ligand and TLR4, as receptor. The most likely TBX2-TLR4 binding complex was selected using the HINT software (Kellogg et al., 1991), available biological information and our own results. In our model, TBX2 could associate through its amino and carboxyl terminus with TLR4 potentially hindering MyD88 and TIRAP binding to TLR4 (Fig. 5).

## 4 Discussion

The efficient and transient intracellular delivery of peptides and polypeptides, collectively named PTDs, allows for reversible regulation of biological processes. As proof of principle, it has been shown that PTDs can serve several uses in therapy. A peptide encompassing NFAT (nuclear factor of activated T cells) inhibitor linked to the cell-permeable sequence Arg10 was used as an immunosuppressive agent, to overcome the undesired toxic effects of calcineurin inhibitors in transplant therapy, enabling successful allogeneic islet transplantation in mice (Noguchi et al., 2004). More recently, a c-Jun N-terminal kinase 1 (JNK1) inhibitory peptide covalently linked at its C terminus to a 10-amino-acid carrier peptide derived from the HIV-TAT sequence, has been shown to improve insulin resistance and glucose tolerance in diabetic mice (Kaneto et al., 2004). In this report, we show that synthetic peptides derived from human proteins of medical interest and containing cationic motifs have the intrinsic ability to translocate across intact extracellular membranes. Similarly to known transduction domains such as TAT, VP22 and Antp, the selected peptides show different extents of cell permeable properties, combined with the ability to function as cargo carriers. In agreement with previous published data, such translocation properties are strongly dependent on arginine and lysine residues (Mi et al., 2000; Park et al., 2002; Mai et al., 2002; Noguchi et al., 2005). Importantly, all peptides chosen for the analysis were capable of entering cells, indicating that although the available collection of five functional transduction peptides identified here is not large enough to rigorously prove our hypothesis, the presence of stretches of arginine and lysine residues is a good marker to predict, before experimental verification, which sequence may function as a PTD. It should to be noted that the choice of the flanking sequences may also affect individual activity.

In addition, to demonstrate intracellular delivery of biologically active polypeptides and to disclose the general therapeutic and technological applications of human peptides containing endogenous cell-permeable motifs, we used an inhibitory peptide entirely derived from the TIRAP adaptor molecule to interfere with the functionality of the TLR4 pathway, as measured by a delay in p38 activation and by the inhibition of cytokine production in LPS challenged cells and mice. Notably, while TBX2 closely reproduced the p38 activation pattern observed in TIRAP knock-out mice, the Antp-TIRAP peptide did not, causing a total inhibition of p38 activation. Although, we cannot rule out that the single amino acid substitution (T- > A) on the TIRAP portion of TBX2 and Antp-TIRAP peptides may contribute to p38 activation, our findings seem to indicate that the presence of the PTD derived from the Drosophila Antp homeodomain induces unwanted biological effects, confirming what had been previously suggested (Toshchakov et al., 2005; Sabroe et al., 2003; Vogel and Fenton, 2003; O’Neill, 2003). The toxicity and immunogenicity of several well known PTDs has been discussed in detail (Trehin R and Mrkle HP 2004). Interestingly, a recent study demonstrated that translocating peptides such as Antp, TAT and nona-arginine (R9) can all induce effects that antagonize or enhance the effects of the cargo being delivered (Fotin-Mleczek et al., 2005). Our results, confirming those obtained with TIRAP knock-out mice, indicate that endogenous translocating peptides such as TBX2 exert a specific function, avoiding unwanted effects and toxicity.

The observation that a fraction of human proteins may contain putative PTDs raises the intriguing possibility that certain proteins may possess the ability to passively translocate through cell membranes, as shown for the human homeoprotein HOXB4 (Amsellem et al., 2003) and the BETA2/NeuroD Protein (Noguchi et al., 2005). Such a notion may also provide a theoretical framework for understanding pathological effects associated with necrosis (as opposed to apoptosis) that commonly occur in tumours, degenerative diseases and chronic inflammatory conditions where cell permeable proteins released by damaged cells could exert an epigenetic pathological effect on the neighbouring ones.

In conclusion, the identification of cell permeable peptides originating from human proteins linked to pathological conditions, offers therapeutical opportunities without the need to use exogenous, often deleterious, PTD sequences to facilitate cellular uptake. This would have the advantage of minimize protein structure artefacts and unwanted biological effects due to the insertion of foreign sequences. These proteins, either in recombinant forms or as synthetic peptides encompassing relevant sequences, may be utilized to regulate protein–protein or protein–DNA interactions, to transiently correct or down regulate abnormal gene function, to interfere with signalling pathways, cell proliferation and to restore genetic defects, therefore facilitating the exploitation of PTD technology in therapy and basic research.
